# Analysis of Multi-Level Simultaneous Driving Technique for Capacitive Touch Sensors

**DOI:** 10.3390/s17092016

**Published:** 2017-09-02

**Authors:** Jong Kang Park, Chang-Ju Lee, Jong Tae Kim

**Affiliations:** 1 College of Information and Communication Engineering, Sungkyunkwan University, Seobu-ro 2066, 16419 Suwon, Korea; jkpark1@skku.edu (J.K.P.); dodo1789@skku.edu (C.-J.L.); 2 System LSI Division, Samsung Electronics, 18448 Hwasung, Korea

**Keywords:** capacitive touch sensor, multi-level Hadamard matrix, simultaneous driving, multi-level driving, code-division multiplexing

## Abstract

The signal-to-noise ratio (SNR) and driving levels of capacitive touch sensors determine the applicability of these sensors to thinner displays and sensor-integrated modules. The simultaneous driving technique has been widely applied to capacitive touch sensors to cope with various types of environmental noise. A Hadamard matrix has been used to determine the driving code and multiplex capacitive signals required to increase the SNR and responsivity of touch sensors. Using multi-level Hadamard matrices, a new driving technique for sensing concurrent capacitive elements across multiple rows of a touch panel was developed. The technique provides more effective design choices than the existing bipolar driving method by supporting a variety of orders of matrices and regular capacity. The required TX voltage can be reduced by applying the Kronecker product for higher orders of simultaneous driving. A system model is presented for multiplexing capacitive signals to extract the SNR of the existing Hadamard matrices as well as one of the proposed multi-level sequences. In addition, the corresponding multi-level drivers and receivers were implemented to verify the theoretical expectations and simulation results of the proposed technique.

## 1. Introduction

The Internet of Things and wearable sensor technologies have accelerated the development of various sensors and their integrated design methodologies. Sensors estimate absolute or relative physical quantities, although their final outputs have different contexts such as the distance, location, and orientation of the target device. Sensors are usually exposed to noise from a variety of sources inside and outside the system. Therefore, noise immunity is a primary metric for quantitatively evaluating sensor systems. In the case of capacitive touch sensors, for overcoming various types of environmental and dynamic noise including display noise, simultaneous driving techniques can achieve a high signal-to-noise ratio (SNR) with a limited response time [1,2]. Because technological advancements continue to yield thinner and more compact panel designs, display noise has become one of the most problematic types of noise that deteriorate the SNR [3,4,5]. For example, if the shielding layer between the display and the touch panel is removed to design more compact and less expensive display modules, the incident display noise for the touch sensor also increases. The SNR decreases with a decreasing thickness of the display and touch panels. High transmission (TX) signals of sensors that are strong to the increased display noise and other external noise sources generate flickers on the display and affect the display’s quality and reliability. TX signal levels can also be constrained in ultrathin panels because of increased parasitic capacitance and power consumption [1,3]. Thus, when placed in close proximity, the display panel and touch sensors compete for their own specifications and performance levels in newer technologies.

This paper presents a new simultaneous TX driving and multiplexing technique for capacitive touch sensors that employs multi-level Hadamard (*M-H*) matrices for communication systems [6,7]. In a capacitive touch panel where many capacitive elements are forming between transparent channels, alternating signals for TX have used to sense the capacitances in a sequential manner. Orthogonal codes used in TX driving fully overlap the sensing time for multiple TX channels and then increase the SNR of the capacitive signal at the receiver [1]. In this work, the theoretical sensor model [8] will be employed to extract SNRs for *M-H* matrices that are applicable to capacitive touch sensors. This attempt provides unified and quantitative views for modeling and evaluating multiplexing techniques for different sensor systems. Unlike two-level bipolar Hadamard (*B-H*) sequences with orders of 2 and 4l [1,9,10,11,12,13,14], the proposed application can theoretically be used to establish any order of orthogonal sequences. Odd number orders of multiple TX lines and the corresponding code sequences can be applied, which yields a better *H* matrix to satisfy the given system requirement for the scan rate or the reporting rate of the sensor to maximize the SNR. Another important feature of these *H* matrices is that regular column sum of the *M-H* matrix and the acquired capacitive signals can be configured due to their capacity efficiency. Although the cost and complexity must be considered in the TX driver design, it can be a competitive option in newer module designs.

In our system model, modulating sequences for orthogonal frequencies [11,15] can also be regarded as another type of orthogonal code for simultaneous driving including multi-level sequences. The orthogonality between sub-carrier frequencies still provides higher SNRs in longer modulating sequences. However, the most frequency-dependent noises (such as the ones from the fluorescent light circuit, battery charger and the display panel) constrain the selectivity of the TX frequencies. Moreover, the slowest TX frequency increases the entire code length and the full scan time of the touch-screen.

This paper is organized as follows. Section 2 presents the system and noise models for the capacitive touch panel as well as its driver and sensing circuits. The system can be formulated as a multiple-input single-output system for communication channels [8]. Section 3 explains how *M-H* sequences for capacitive sensors can be constructed using circulant matrices. It also presents a theoretical analysis and the variants of basic *M-H* matrices. Section 4 contains the numerical results of the simulations and experiments, including an analytical comparison between the conventional *B-H* and *M-H* matrices. Finally, the paper is concluded in Section 5.

## 2. Capacitive Touch Sensing System

### 2.1. System Model

A theoretical model for capacitive touch sensors can be derived from a multiple input multiple output (MIMO) system that is stimulated by orthogonal active signals [8]. In a two-dimensional (2-D) capacitive touch panel with NT TX and NR reception (RX) channels, we first consider a row vector of the capacitance matrix is assumed to be $c_{j}=\left\{c_{1j},c_{2j},\cdots ,c_{NTj}\right\}$. Let $N_{code}$ and $N_{chip}$ be the code length and the chip length for code division multiplexing, respectively. Assume that $N_{chip}$ should be sufficiently lengthy to support the settling time of the amplifier and the input network. In H with NT rows and $N_{code}\times N_{chip}$ columns where $NT\leq N_{code}\times N_{chip}$, the inner product of any two different row vectors is zero. Then, the signal $X_{j}$ passing through a capacitance vector $c_{j}$ can be decoded as $r_{j}=\left\{r_{1j},r_{2j},\cdots ,r_{NTj}\right\}$, as follows:

(1) $$\begin{array}{ccc} r_{j} & = & X_{j}H^{⊤}=\left(\alpha V_{TX}H+Z_{j}\right)H^{⊤}\\ & ≃ & \alpha V_{TX}\cdot N_{code}\cdot N_{chip}\cdot I_{NT} \end{array}$$

where α is the voltage-to-voltage conversion parameter from the driver to the receiver circuit. It will be determined by the input network including $c_{j}$, the feedback components of the amplifier, and the gain of the post-stage filters. $V_{TX}$ is the reference voltage level for generating the TX driving sequence in conjunction with H matrix. $Z_{j}$ is an error and noise vector at receiver *j* originating from different path characteristics and noise components. In [8,16], it was additive to the final decoded output for simplicity. To extract the SNR based on the length of spreading codes (H), it is added to the capacitive signals $X_{j}$. In this paper, only the dynamic components for $Z_{j}$ was considered. If $Z_{j}$ can be minimized as a zero vector, after being decoded via multiplication by $H^{⊤}$, H is then converted to $\left(N_{code}\times N_{chip}\right)\cdot I_{NT}$, where $I_{NT}$ is the NT×NT identity matrix. For the construction of H satisfying (1), entire or partial rows of the Hadamard matrix $H^{*}$ can be utilized, as follows:

(2) $$H=H^{*}⊗J_{1,N_{chip}}⊗\left[\begin{array}{cc} -1, & 1 \end{array}\right]$$

where ⊗ is the Kronecker product added to the alternating current (AC) signal to detect the capacitive element, and $J_{1,N_{chip}}$ is an $1\times N_{chip}$ matrix of ones. The number of columns in $H^{*}$ is defined as $N_{code}$. It is assumed that $N_{chip}$ is predetermined by analog and digital sensing circuits for the given sensor device. We define $T_{chip}$ as a unit chip period; thus, the length of the total code sequence is $N_{code}\times N_{chip}\times T_{chip}$.

**Figure 1:**
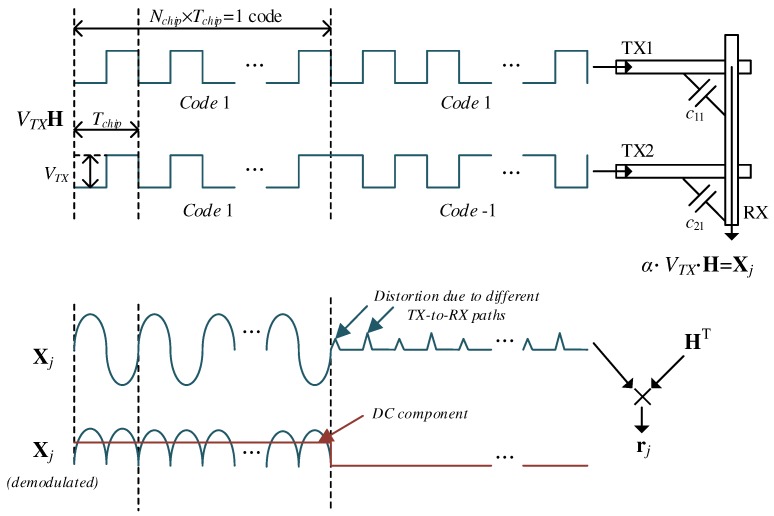
Example of two-channel simultaneous driving and sensing.

Figure 1 shows an example of two-channel simultaneous driving and sensing of capacitive signals in a touch panel based on (1) and (2). For simplicity, $H^{*}$ is chosen as the Hadamard matrix with order two. To pass the capacitive elements $c_{11}$ and $c_{21}$, it is expanded to H by (2) and then used to generate actual driving signals, $X_{j}=\alpha \cdot V_{TX}\cdot H$ in (1). The AC signals for TX1 and TX2 propagate along $c_{11}$ and $c_{21}$, respectively. After multiplication by the operational gain α at receiver *j*, the acquired charges are detected by the identity, Q=CV. Because there are two codes per TX channel, the detected charges are represented as two sample voltages, which are equivalent to the integrated current in the circuit capacitance during each $N_{code}\times N_{chip}$ period. These two samples are then decoded using $H^{⊤}$. Ideally, spread sequence $X_{j}$ are perfectly aggregated by H. However, small distortion on $X_{j}$ exists due to the different path delays and signal gains between TX1-RX and TX2-RX.

### 2.2. Random Noise Components for Capacitive Touch Sensors

Propagation characteristics for TX-to-RX paths and the crosstalk noise for constant driving codes in H cause static errors in sensing target capacitances. These static errors can usually be corrected using the delay controls of the driving codes and value compensation on the receiver side due to the static nature that is determined by the touch panel structure and circuit implementation. Conversely, random noise components of sensing values result in touch location jitters and detection failures. These dynamic errors primarily depend on the SNR of the capacitive signal acquired by the analog front end of the receiver. For a large in-band noise, we can change the modulation frequency of the TX [17]. To suppress the remaining dynamic errors, in the software of the touch sensor, the spatial and temporal properties of the TX-to-RX values on the entire touch panel can be utilized at a late processing stage [18,19,20].

**Figure 2:**
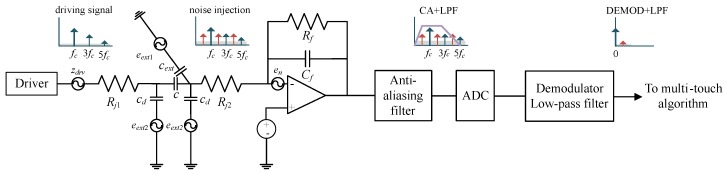
Signal processing flow and possible noise components.

Figure 2 shows a signal processing flow of a capacitive touch sensor and summarizes the relevant dynamic noise components. A driver generates modulated TX signals with frequency $f_{c}$. In conjunction with $f_{c}$ and its harmonic components, several noise sources from drivers, resistances, amplifiers, coupling noise through fingers and display panels can be injected and propagated through the capacitive element *c* and the charge amplifier (CA). These noises are attenuated by the frequency responses of CA and an anti-aliasing filter. After de-modulating with $f_{c}$, the capacitive signal representing *c* appears at zero frequency. Let $z_{ij}$ in $Z_{j}$ be the root-mean-square (RMS) value of the total noise at the *i*-th spread code and *j*-th receiver circuit. Then, $z_{ij}$ for the number of concurrent driving channels *n* can be defined as follows:

(3) $$\begin{array}{ccc} z_{ij}^{2} & = & \sum_{k}^{n}z_{drv\_kj}^{2}\cdot \int {\left|A_{v}\left(f\right)\right|}^{2}{\left|A_{LPF}\left(f\right)\right|}^{2}df+\sum 4k_{B}TR_{j}\int {\left|A_{R_{j}}\left(f\right)\right|}^{2}{\left|A_{LPF}\left(f\right)\right|}^{2}df\\ & & +4k_{B}TR_{f}\int {\left|A_{LPF}\left(f\right)\right|}^{2}df+\sum z_{ext}^{2}+z_{amp\_j}^{2} \end{array}$$

where $z_{drv\_kj}$, $z_{ext}$, and $z_{amp\_j}$ are the RMS noise level for driver circuit *k* appearing at receiver *j*, the in-band external noise component and the amplifier noise in receiver *j*, respectively. These are categorized into purely random or correlated sources of electronic noise [21]. $A_{v}$ which is the closed loop gain of CA [22], adjusts all noise components appearing at the input of the amplifier by feedback components $R_{f}$ and $C_{f}$. $R_{j}$ represents a series resistance on a TX-to-RX path arriving at receiver *j*. On the other hand, as a feedback resistor of CA, $R_{f}$ can be changed by a gain configuration. By employing Boltzmann’s constant $k_{B}$ and the absolute temperature *T*, we obtain two thermal noise factors that are affected by the gain of the receiver for $R_{j}$ ($A_{R_{j}}$), and the gain of the anti-aliasing filter ($A_{LPF}$), as shown in (3). In (3), $z_{amp\_j}$ can be determined by the input noise voltage of the amplifier [23,24,25], as follows:

(4) $$z_{amp\_j}=\int \left|1+\frac{Z_{in}}{Z_{f}}\right|\cdot \left|e_{nj}\left(f\right)\right|\cdot \left|A_{LPF}\left(f\right)\right|df$$

where $e_{nj}$ is the spectral density of the noise voltage defined in the specification of amplifier *j* [25], and $Z_{in}$ denotes the impedance of the input network of the amplifier including *c*. $Z_{f}$ is the impedance of the feedback path, which is composed of $R_{f}$ and $C_{f}$. Although the modulation frequency ($=1/T_{chip}$) in (2) is carefully chosen to avoid the main frequency of external noise sources [17], the remaining noise level of $z_{ext}$ still exists after de-modulation via multiplication by $H^{⊤}$ in (1) due to the limited bandwidth of the touch panel. Here, $z_{ext}$ can be expressed in terms of the spectral density of noise $e_{ext}$.

(5) $$z_{ext}=\frac{1}{4}\int \left|e_{ext}\left(f-f_{c}\right)+e_{ext}\left(f+f_{c}\right)\right|\cdot \left|A_{ext}\left(f\right)\right|\cdot \left|A_{LPF}\left(f\right)\right|df$$

where $1/2\cdot \left\{e_{ext}\left(f-f_{c}\right)+e_{ext}\left(f+f_{c}\right)\right\}$ represents an amplitude modulation of $e_{ext}$ in the frequency domain. $f_{c}$ is a modulation frequency that is implicitly defined in (2) where [−1, 1] modulates each code in a row of H. $e_{ext}$ includes the noise density injected into the touch channel from a touching object with $c_{ext}$ or from the display panel with $c_{d}$. Note that the magnitude responses of $A_{ext}\left(f\right)$, $A_{v}\left(f\right)$, $A_{R_{j}}\left(f\right)$ are proportional to $\left|Z_{f}\right|$ while their input impedances are invariant. Thermal noise from $R_{f}$ should also be determined by $\left|Z_{f}\right|$. Thus, except for $z_{amp\_j}$, which contains an inverse proportional term to $\left|Z_{f}\right|$, most noise components in (3) are directly scaled by the feedback gain control of CA. If the conversion parameter $\alpha \propto \left|A_{v}\left(f_{c}\right)\right|\propto \left|Z_{f}\right|$, we can approximately say that $z_{ij}\propto \alpha$.

## 3. Multi-Level Sequences for Capacitive Sensors

**Figure 3:**
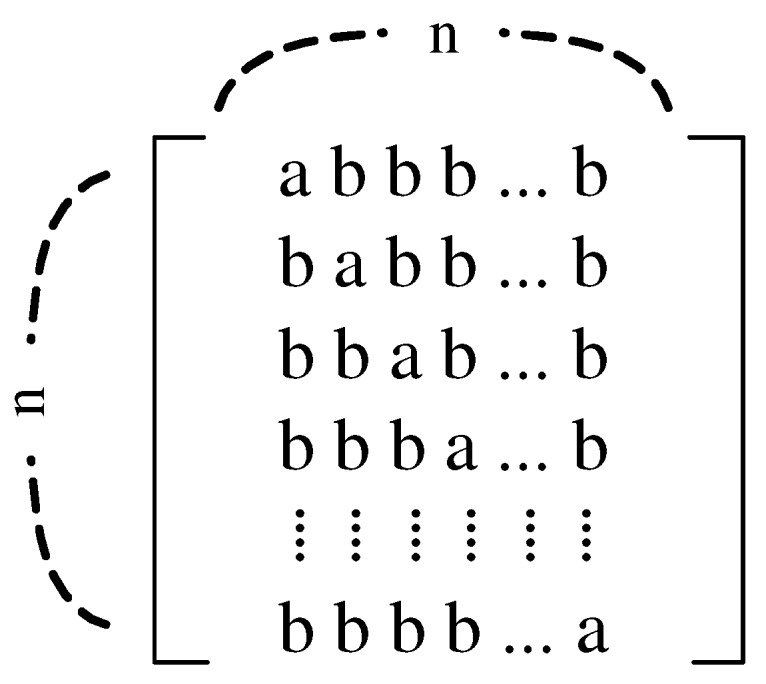
Circulant matrix for constructing multi-level *H* matrices.

Consider the *M-H* matrix applicable to $H^{*}$ in (1). A circulant matrix can be used to form *M-H* matrices [6]. Suppose that the first row of the *M-H* matrix is (abb⋯b), where *a* and *b* are real numbers. The remaining rows are represented in the form of (bab⋯b), (bba⋯b), ⋯, and (bbb⋯a), as shown in Figure 3. The code values *a* and *b* in $H^{*}$ define the relative TX driving voltage levels in a sequence, and the column sum of $H^{*}$ represents the maximum input range of the receiver, as well as the peak swing levels in the corresponding TXs-to-RX paths of a touch screen. We formulate two properties for M-H matrices as follows:

(6) $$\begin{array}{cc} \mathrm{Orthogonality}: & 2ab+\left(n-2\right)b^{2}=0 \end{array}$$

(7) $$\begin{array}{cc} \mathrm{Max}.\mathrm{column}\;\mathrm{sum}: & m=a+\left(n-1\right)b<\frac{M}{\alpha V_{TX}} \end{array}$$

where *n* is the order of the *M-H* matrix. *M* is the maximum capacity for capacitive signals limited by the sensing circuit. This implies that the effective signal is only accepted in [-*M*, +*M*] V. In real application, the inequality in () can be satisfied by controlling $\left|Z_{f}\right|$ to adjust α. Using (6) and (), the following is obtained:

(8) $$\begin{array}{ccc} a & = & m(2/n-1)\\ b & = & 2m/n \end{array}$$

which are functions of *m* for the given *n*. In (1), the multiplication of $H^{⊤}$ at receiver *j* does not increase the signal gain against the noise component while $X_{j}=\alpha V_{TX}H$ determines the capacitive signal levels. It is assumed that $c_{j}$ consists of the same constant *c*. If every element $x_{ij}\in X_{j}$ is replaced with the Gaussian random variables $N(\mu_{x},z_{x}^{2})$ with a mean $\mu_{x}$ and the variance $z_{x}^{2}$, Equation (1) can be rewritten as:

(9) $$r_{j}=\left[\begin{array}{c} N\left(\mu_{1j},z_{1j}^{2}\right),N\left(\mu_{2j},z_{2j}^{2}\right),\cdots ,N\left(\mu_{ij},z_{ij}^{2}\right),\cdots ,N\left(\mu_{nj},z_{nj}^{2}\right) \end{array}\right]\cdot H^{⊤}.$$

Then, the received signal $r_{ij}$ for the *i*-th driver and *j*-th receiver can be decoded as:

(10) $$r_{ij}=\left[\begin{array}{c} N\left(\mu_{1j},z_{1j}^{2}\right),N\left(\mu_{2j},z_{2j}^{2}\right),\cdots ,N\left(\mu_{ij},z_{ij}^{2}\right),\cdots ,N\left(\mu_{nj},z_{nj}^{2}\right) \end{array}\right]\cdot h_{i}^{⊤}$$

where $h_{i}$ denotes the *i*-th row vector in H. In regular *H*-like *M-H* matrices, all the elements in X are identical. If $r_{ij}$ is assumed to be $r_{x}$, Equation (10) can be expanded by using the same random variable $N(\mu_{x},z_{x}^{2})$:

(11) $$\begin{array}{ccc} r_{ij} & = & aN\left(\mu_{x},z_{x}^{2}\right)+bN\left(\mu_{x},z_{x}^{2}\right)+\cdots +bN\left(\mu_{x},z_{x}^{2}\right)\\ & = & N\left(a\mu_{x},a^{2}z_{x}^{2}\right)+N\left(b\mu_{x},b^{2}z_{x}^{2}\right)+\cdots +N\left(b\mu_{x},b^{2}z_{x}^{2}\right)\\ & = & N\left(\alpha V_{TX}\left\{a^{2}+\left(n-1\right)b^{2}\right\},a^{2}z_{x}^{2}+\left(n-1\right)b^{2}z_{x}^{2}\right) \end{array}$$

where $\mu_{x}=m\cdot \alpha V_{TX}=\left\{a+\left(n-1\right)b\right\}\cdot \alpha V_{TX}$. The property is applied for the sum of the independent random variables [26] to (11). Then, the SNR for $r_{ij}$ can be defined as:

(12) $$\begin{array}{ccc} \mathrm{SNR}(r_{ij}) & = & \alpha V_{TX}\cdot \frac{a^{2}+\left(n-1\right)b^{2}}{\sqrt{a^{2}z_{x}^{2}+\left(n-1\right)b^{2}z_{x}^{2}}}\\ & = & \alpha V_{TX}\cdot \frac{\sqrt{a^{2}+\left(n-1\right)b^{2}}}{z_{x}}. \end{array}$$

By substituting (8) into (12), the following is obtained:

(13) $${\mathrm{SNR}}_{MH}\left(r_{ij}\right)=\alpha V_{TX}\cdot \frac{m}{z_{x}}.$$

Similarly, according to (10), for the bipolar *H* matrix where $|h_{ij}|=1$,

(14) $$\begin{array}{ccc} r_{ij} & = & h_{1j}N\left(\mu_{1j},z_{1j}^{2}\right)+h_{2j}N\left(\mu_{2j},z_{2j}^{2}\right)+\cdots +h_{nj}N\left(\mu_{nj},z_{nj}^{2}\right)\\ & = & N\left(h_{1j}\mu_{1j},h_{1j}^{2}z_{1j}^{2}\right)+N\left(h_{2j}\mu_{2j},h_{2j}^{2}z_{2j}^{2}\right)+\cdots +N\left(h_{nj}\mu_{nj},h_{nj}^{2}z_{nj}^{2}\right)\\ & = & N\left(h_{1j}\mu_{1j}+h_{2j}\mu_{2j}+\cdots h_{nj}\mu_{nj},h_{1j}^{2}z_{1j}^{2}+h_{2j}^{2}z_{2j}^{2}+\cdots h_{nj}^{2}z_{nj}^{2}\right)\\ & = & N\left(\alpha V_{TX}\cdot n,z_{1j}^{2}+z_{2j}^{2}+\cdots +z_{nj}^{2}\right) \end{array}$$

Therefore, the SNR for a bipolar *H* matrix can be defined as:

(15) $${\mathrm{SNR}}_{BH}\left(r_{ij}\right)=\alpha V_{TX}\cdot \frac{n}{\sqrt{z_{1j}^{2}+z_{2j}^{2}+\cdots +z_{nj}^{2}}}.$$

when $z_{ij}≃z_{x}$ from the assumption, Equation (15) can be further simplified as,

(16) $${\mathrm{SNR}}_{BH}\left(r_{ij}\right)≃\alpha V_{TX}\cdot \frac{\sqrt{n}}{z_{x}}.$$

When considering the SNR for the touch event, $r_{ij}$ should be replaced by $\left|\Delta r_{ij}\right|$ in (13) and (16). α decreases by the increase of the input impedance of CA due to a touch event. A typical definition for SNR contains $\left|\Delta r_{ij}\right|$ instead of $r_{ij}$ [1,27]. However, in this work, both SNR($\left|\Delta r_{ij}\right|$) and SNR($r_{ij}$) definitions are employed. For equivalence to the existing definition [1,27], the numerator in (12) and (15) corresponds to the mean value of the signal samples, and its additive noise $z_{x}$ can be replaced by the standard deviation of them.

Consequently, the acquired signal for one sequence in the *M-H* matrix is proportional to *m*, which is identical to the sum of the TX levels in a column of H. For regular and Bush-type *B-H* matrices with $n=m^{2}$, whose column sums and row sums are all equal to *m* [28], it can be concluded that ${\mathrm{SNR}}_{MH}\left(r_{ij}\right)≃{\mathrm{SNR}}_{BH}\left(r_{ij}\right)$, according to (13) and (16). These special type *H* matrices are rare, and their orders are constrained as $4l^{2}$, where *l* is a positive integer. For an irregular *B-H* matrix with $n<m^{2}$, ${\mathrm{SNR}}_{MH}\left(r_{ij}\right)>{\mathrm{SNR}}_{BH}\left(r_{ij}\right)$, where $z_{ij}=z_{x}$.

**Figure 4:**
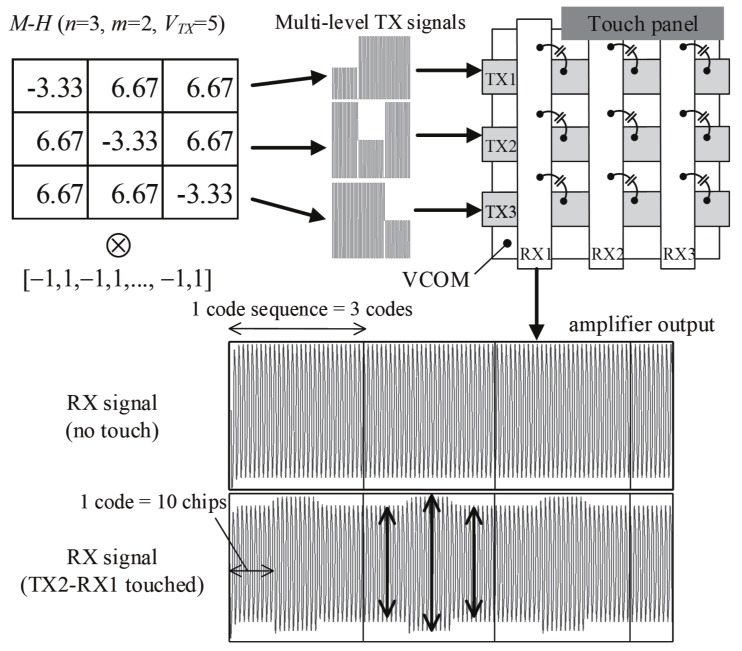
Multi-level TX sequence and the aggregated RX signal at the receiver.

Figure 4 provides an example of H with $N_{chip}$ = 10 in the form of an *M-H* matrix and the one corresponding output of the receiver with *n* = 3 and *m* = 2. The maximum column sum is identical to that of a typical Walsh-Hadamard (*W-H*) matrix with n=2. Both the column sum and the row sum are equal to *m* in such *M-H* matrices. When a touch event decreases the capacitance across TX2 and RX1, the first and third receiving signals for the second TX sequence [6.67, −3.33, 6.67] are decreased. Conversely, the second signal is increased due to the negative sign of the second code.

The orders of *M-H* matrices can be constructed so that a given time slot for sensing can be fully utilized. To maximize the capacitive signals, the length in multiples of the code and chip sequence should match the required time slot [1]. This period is usually constrained by the scan rate or a reporting rate corresponding to the response time of the sensor system. Because typical *H* matrices exist in 2 and 4l-length squares, it is difficult to only adjust the multiples of the columns in the *H* matrix to increase the signal gain. For example, *H* matrices with orders of 2 and 4l do not fit time slots 3, 5, 6, 7, 9, and 10. Conversely, the suitability of *M-H* matrices with odd and even orders for the increased number of columns can be adjusted by removing unnecessary rows or by combining two or more *M-H* matrices.

**Figure 5:**
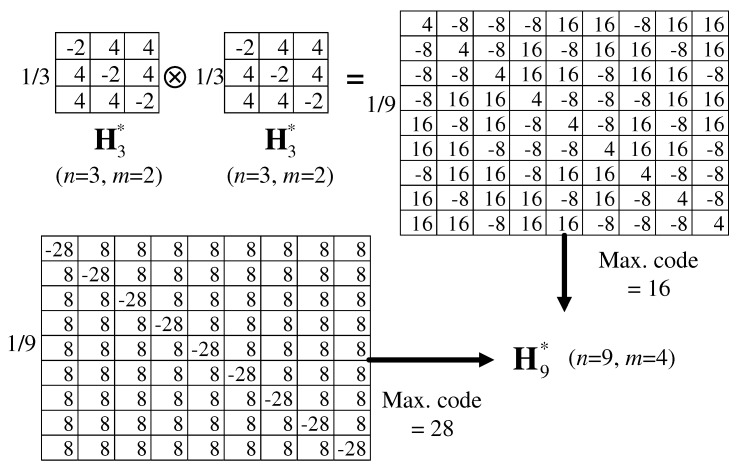
Expansion of an *M-H* matrix using Kronecker product.

Multi-level $H_{n}$ matrices of order *n* can be expanded using the Kronecker product ⊗. Let $H_{A}$ and $H_{B}$ be the *M-H* matrices satisfying (6)–(8). From $H_{C}=H_{A}⊗H_{B}$, $m_{C}=m_{A}\cdot m_{B}$ is derived. By using an expanded matrix $H_{C}$, the elements in $H_{C}$ are better distributed in their absolute values and then the maximum code that represents the peak-to-peak TX driving level can be made lower than that of the original *M-H* matrix directly constructed by (6) and (). As shown in Figure 5, the $H_{9}^{*}$ obtained by $H_{3}^{*}⊗H_{3}^{*}$ decreases the maximum TX voltage by 43% compared with the original $H_{9}^{*}$, whereas the column sum *m* is identical in both cases. Additionally, the Kronecker product for two *M-H* codes reducing the maximum TX voltage eases the power requirement for the individual driver circuit.

## 4. Results and Discussion

### 4.1. Simulation Results with Constant $z_{x}$

In this subsection, the conventional *W-H* matrix, the tuned *H* matrix, which is described in [1], and the proposed *M-H* matrices are compared when the signal capacity of the receiver is limited. To maximize the SNR in *M-H* and *B-H* matrices, we can scale *m* up to $M/\alpha V_{TX}$, as defined in ().

**Figure 6:**
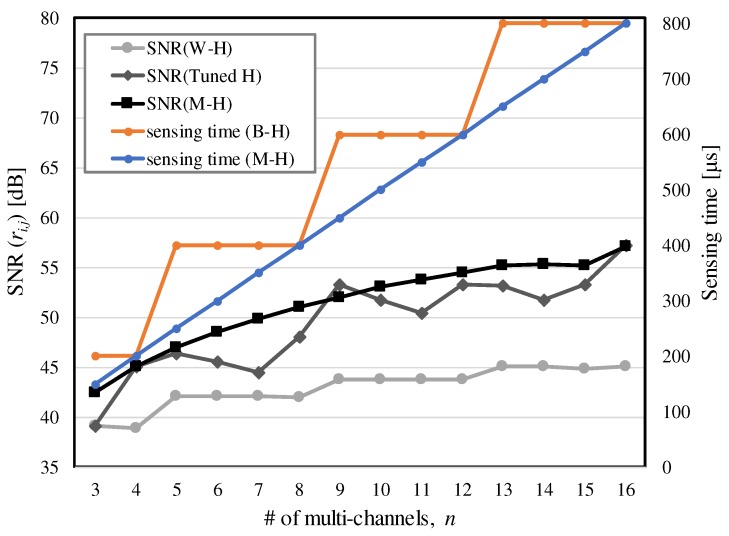
Comparison of signal acquisition properties for the identical *m* between *B-H* [1,10,12,13,14] and proposed *M-H* matrices where $\alpha V_{TX}=350$ mV and $z_{x}=7.7$ mV.

Figure 6 summarizes the capacitive signal gain and sensing time for a sequence, sampled at the output of the decoder for the corresponding *H* matrix, where a code consists of 10 chips with a 200 kHz driving frequency. The transfer functions of the TXs-to-RX network and CA were extracted using the circuit design and measurement data for a bar-type touch panel. A total of 10,000 runs of a Gaussian random noise simulation was iterated to obtain the significant digits. Simulated results for SNRs could be verified by (13) and (16). In this simulation, the TX voltage levels of the *M-H* matrices and tuned *H* matrices were scaled according to the column sum of the *W-H* matrix for a given *n*. The TX levels were adjusted so that the maximum column sum was equal to the same target *m* (= *n*) for all *H* matrices for a given number of multi-channels or codes. Kronecker products were used in extracting *M-H* matrices (*n* = 8, 9, 10, 12, 14, 15, 16) to reduce the maximum TX level. Even if there are no sensing time constraints, the *M-H* matrices yielded higher SNRs than the other *H* matrices, except in the nine-channel case. For the tuned *H* matrix with *n* = 9, the maximum column sum (*m*) can be decreased to 3 [1]. It shows *n* = $m^{2}$, as in the *M-H* matrix. Since its code length (= 12) is greater than 9, the resulting SNR can be higher than that of the *M-H* matrix. However, most irregular *B-H* matrices in $n<m^{2}$ showed lower SNRs, as stated in Section 3. The conventional *B-H* sequences including the tuned *H* matrices, require two or 4l length of codes. The *M-H* matrices give the same code length for a given *n*. As shown in Figure 6, this advantage results in a shorter sensing time than the *B-H* matrices. The current model assumes a fixed number of cycles ($N_{chip}\times N_{code}$) in an acquisition of capacitive signals. However, if the receiver circuit will be able to support the integration of the charges for every cycle, the SNRs of the *M-H* codes in Figure 6 will be more improved while their sensing times are increased to those of the tuned *H* matrices.

When improving the SNR, it is inevitable that the total energy consumption for concurrent TX driving increases. This is not due to the type of code, but to the driving levels and the resulting SNR. The code value and $V_{TX}$ determine the peak-to-peak swing level of the TX. Even if the polarity of the code sequence still affects the number of transitions during the driving, its power consumption is a function of squared code [1]. Let $n_{MH}$ and $n_{BH}$ be the orders of the *M-H* and *B-H* codes, respectively. If the target code length is defined as $n_{MH}$, because of its universality for orders, the sums of squared codes for the *B-H* and *M-H* matrices can be defined as $n_{MH}\cdot n_{BH}$ and $n_{MH}\cdot m^{2}$, respectively. As stated in Section 3, $n_{BH}$ determines the SNR of the *B-H* codes while *m* defines the SNR of the *M-H* codes. Thus, both energy consumption and the SNR for the *M-H* case will be higher than the ones of irregular *B-H* matrices where $n_{BH}$ < $m^{2}$. Otherwise, if $n_{BH}$ = $m^{2}$, SNRs and energy consumptions are nearly the same in the two cases.

### 4.2. $z_{x}$ Variation with Respect to *n*

**Figure 7:**
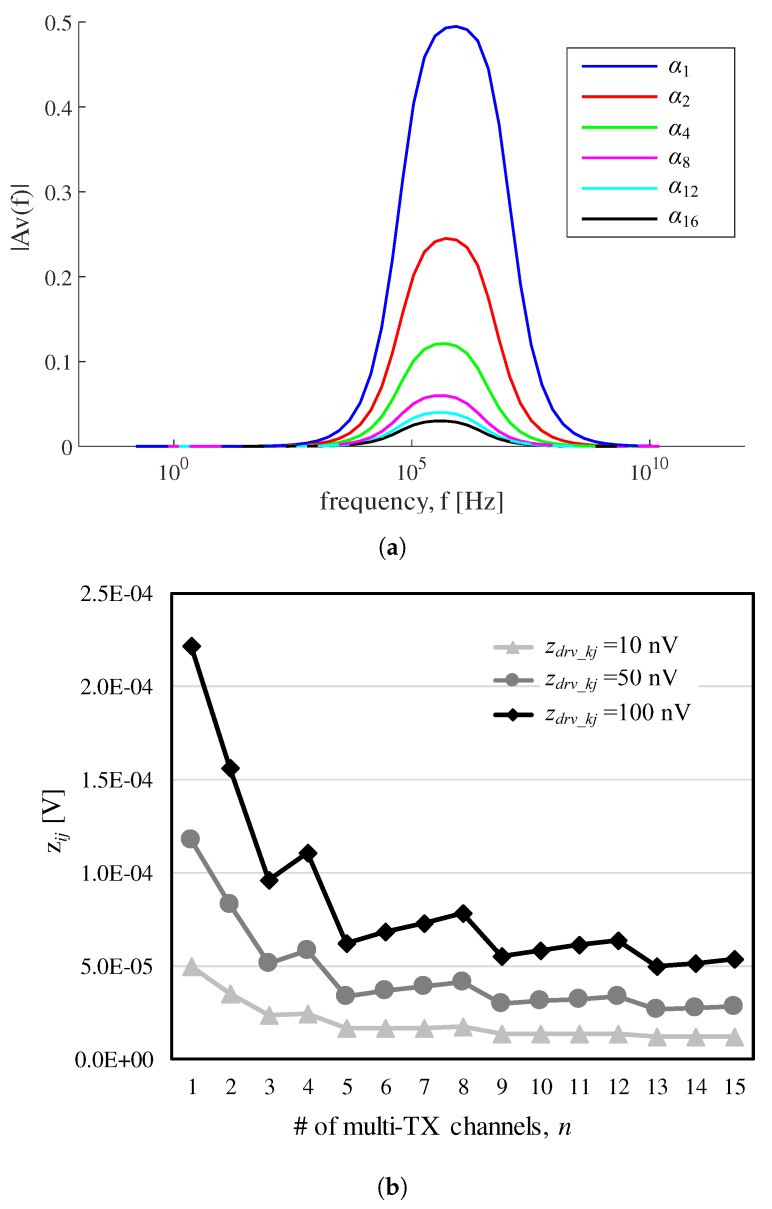
(**a**) $A_{v}\left(f\right)$ for different gain sets of CA, (**b**) Variation of $z_{ij}$ with respect to *n* without considering $z_{ext}$.

By observing the inequality in (7), the converted noise $z_{ij}$ in (3) can practically decrease in higher simultaneous TX channels under the assumption with small $z_{drv\_kj}$ and small $z_{amp\_j}$. The increase in the number of simultaneous TX channels should consider the maximum capacity (*M*) of the receiver so that α should be scaled down not to exceed *M* in (7). The column sum *m* in an *H* matrix determines the maximum capacitive signal level and increases as the number of concurrent driving channels *n* increases. Therefore, $A_{v}\left(f\right)$ must be reduced to satisfy (7). In this regard, $C_{f}$ should be increased in order to decrease α and eventually, to not exceed *M*. To maintain the same bandwidth in $R_{f}\cdot C_{f}$, $R_{f}$ must be reduced accordingly [1]. For example, when $R_{f}=500$ kΩ and $C_{f}=4$ pF are initially chosen to detect c=2 pF, for the half gain to this initial set, $R_{f}=250$ kΩ and $C_{f}=8$ pF should be configured. As shown in Figure 7a, there can be different $R_{f}$ and $C_{f}$ configurations to support *n* and corresponding magnitude responses. For simplicity, the pair of $\left(R_{f},C_{f}\right)$ is represented by $\alpha_{n}=\left(500\;k\Omega /n,4\;\mathrm{pF}\times n\right)$.

The difference in the gain magnitude between any distinct sets of $\left(R_{f},C_{f}\right)$ are maintained for all frequency samples. Figure 7b shows RMS plots of the noise components with respect to *n* except for the external noise and amplifier noise. Assume that the individual driver noise $z_{drv\_kj}$ varies from 10 nV to 100 nV. Each resistance value around *c* in the touch panel is set to 1 kΩ. For *n* = 3, 5 to 8, 9 to 12 and 13 to 15, we configured the closed loop gain as $\alpha_{4}$, $\alpha_{8}$, $\alpha_{12}$ and $\alpha_{16}$, respectively. All noises that occur prior to CA including $z_{drv\_kj}$ and thermal noise from $R_{j}$ are directly attenuated by $A_{v}\left(f\right)$ and $A_{R_{j}}\left(f\right)$. As $R_{f}$ is scaled down at a higher *n*, the thermal noise component for $R_{f}$ in (3) must also be reduced. In cases with *n* = 2, 3, 5, 9 and 13, $z_{ij}$ is instantly suppressed by this property. For $z_{drv\_kj}^{2}$ with same gain configuration in (3), the aggregated driving noise power increases by *n* times. Thus, $z_{ij}$ is slightly increased when *n* = 4, 6 to 8, 10 to 12 and 14 to 15. The increase in $z_{ij}$ is clearer in $z_{drv\_kj}=100$ nV than in $z_{drv\_kj}=10$ nV. This effect also indicates that a large driving noise can degrade the theoretical SNRs defined in (13) and (15).

**Figure 8:**
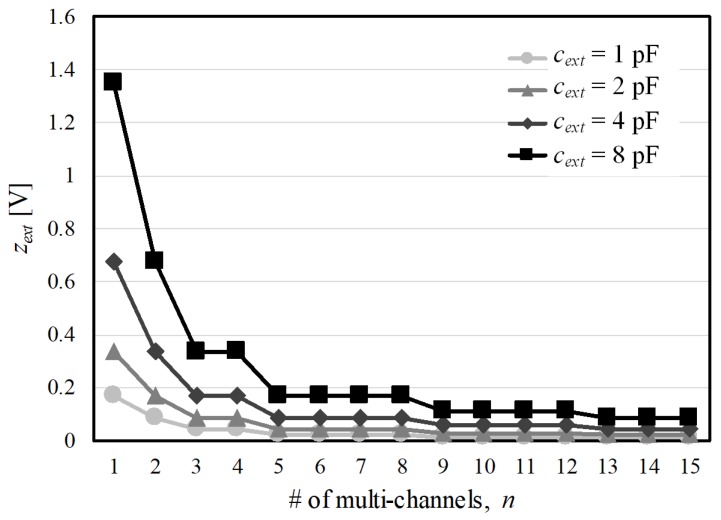
Reduction in the external noise with respect to the number of TX channels and for different gain sets in $f_{c}=380$ kHz, $e_{ext}\left(f\right)=\delta \left(f-200k\right)$.

In contrast, $z_{ext}$ depends on the amount of noise-induced charges that pass through the capacitive elements from a touching object and from the display panel. In (5), these noises are further modulated by $f_{c}$. Therefore, with the low-pass filter in the succeeding process, a larger difference between the fundamental frequencies of the noises and $f_{c}$ yields less in-band noise $z_{ext}$. For incident noise through fingers, more noisy charges are injected, as a larger conducting object overlays the TX and RX channels. In practice, $z_{ext}$ is often significantly distorted by the difference between the sensor ground and the real ground for noise sources (i.e., earth level). On the other hand, display noises are induced by display drivers and their common electrode (VCOM) regardless of touch events [3]. Different display images, vertical structures and driving methods result in various negative effects to the over-layered and embedded capacitive sensors in display modules. Figure 8 shows the change in the modulated external noise with respect to *n* and the coupling capacitance $c_{ext}$ that is proportional to the size of the noisy area. A higher *n* requiring a decrease in $\left|A_{v}\left(f\right)\right|$ reduces the incident noise. $z_{ext}$ increases entirely when $c_{ext}$ covers a larger part of conducting channels ranging from $c_{ext}=1$ pF to $c_{ext}=8$ pF. However, a higher *n* requiring a low $\left|A_{v}\left(f_{c}\right)\right|$ still reduces the external noise. The result shows that $z_{ext}$ in a fixed $c_{ext}$ is proportional to α, which was set by the one of configuration sets in Figure 7a.

**Figure 9:**
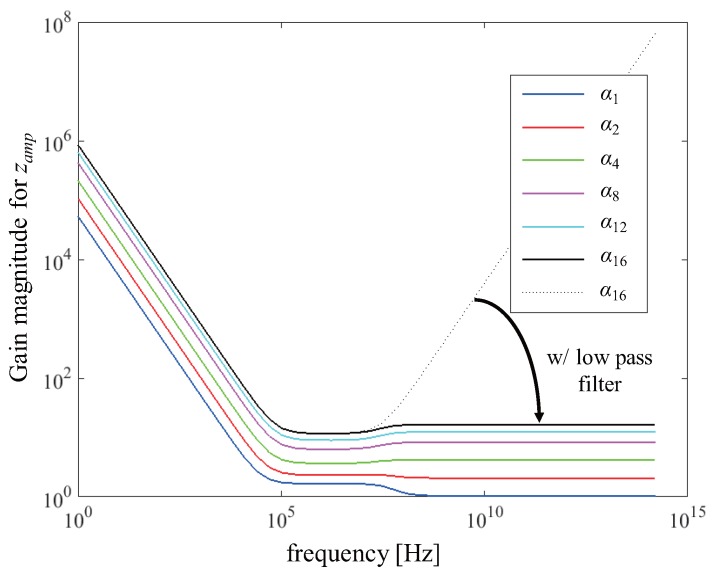
Frequency response for $z_{amp\_j}$.

In summary, $z_{ij}$ with a relatively small $z_{drv\_kj}$ can eventually be decreased due to an obligate use of lower α at a higher *n*. However, unlike the overall relation between $z_{ij}$ and α, $z_{amp\_j}$ for CA would be increased according to (4). As shown in Figure 9, increasing *n* results in an excess of $z_{amp\_j}$ when even higher frequency components are limited by post-stage low-pass filters. The dotted line and solid line show the frequency responses of *n* = 16 with and without a low-pass filter, respectively. Below a frequency of 10 MHz, $z_{amp\_j}$ is inversely proportional to α. This clearly shows $z_{ij}$ can increase in a low α, if $z_{amp\_j}$ dominates $z_{ij}$. Theoretical SNRs for simultaneous driving derived in (13) and (16) must be degraded in that case. Therefore, to improve SNR, it would be advantageous to decrease the maximum column sum of the *H* matrix satisfying *M* and eventually not to decrease α at a higher *n*.

### 4.3. Implementation

**Figure 10:**
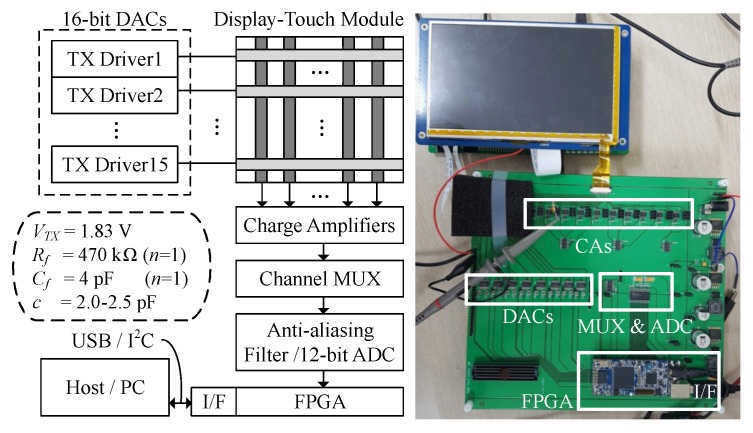
Experimental setup.

To verify the theoretical noise immunity of the proposed *M-H* sequences, multi-level drivers that support simultaneous TXs were implemented. A transparent touch-screen panel embedded display module with 15 TX channels was tested using the drivers, receiver circuits, and a controller in a field-programmable gate-array (FPGA) device. Figure 10 shows the block diagram and the implemented hardware for the experimental setup. The driver circuits implemented by eight 2-channel 16-bit digital-to-analog converters (DACs) generate simultaneous multi-level waves to stimulate the capacitive touch panel and were controlled by logic circuits that were configured in the FPGA device. Ten parallel CAs, a channel multiplexer, and a 12-bit analog-to-digital converter (ADC) were implemented in a time-shared receiver, effectively establishing 10 RXs. The charges crossing the capacitive elements were first converted to the voltage signal by CA. After passing through an anti-aliasing filter and ADC, a digitized signal was sent to the FPGA and then de-modulated and de-spread by $H^{⊤}$, as defined in (1). The 2-D 15 × 10 capacitance matrix can be monitored by the host machine through either of a universal serial bus or an I^2^C interface. This test setup can be re-configured for different codes and panel structures by changing only $R_{f}$, $C_{f}$, and the embedded memory elements located in the FPGA. Multi-level concurrent driving signals can be finely adjusted by 16-bit DACs configurable at the host PC. Different display images could also be tested via the commercial Android platform and its debug interface.

Table 1 summarizes the SNRs obtained by a single acquisition for several *M-H* matrices and the conventional *B-H* matrices [1,10,12,13,14]. $\left|\Delta r_{ij}\right|$ for the touch event is equivalent to 20% of $r_{ij}$. Received signals for $r_{ij}$ and $\left|\Delta r_{ij}\right|$ are the average values of a total of 256 samples where $N_{chip}$ = 15. $z_{x}$ calculated from the standard deviation of 256 received signals when a touch event exists. Specifically, a regular *B-H* matrix or a tuned *H* matrix was selected for the case in the case of n=4 and m=2. Errors between observed SNRs for $r_{ij}$ and the one for the analytical model using (3), (12), and (15) and the fitting error are listed in the rightmost column. In practice, supply noises appearing at discrete ICs further degrade the SNR of the acquired signal [25]. We tried to fit the major components of (3) by the measurement. To characterize the noise variation dependent on the gain and the number of driving signals, $z_{x}$ in (3) can be further simplified into the following relation:

(17) $$z_{x}^{2}≃n\alpha_{r}^{2}A+\alpha_{r}^{2}B+1/\alpha_{r}^{2}C$$

where α = $\alpha_{r}\cdot \alpha_{1}$. $\alpha_{r}$ and $\alpha_{1}$ denote the relative gain and signal gain at *n* = 1, respectively. *A*, *B*, and *C* represent the scaled components from the driver noise, in-circuit noise including thermal noise, and the amplifier noise defined in (3), respectively. As we explained in Section 4.2, *A* and *B* are directly attenuated by the closed loop gain of the amplifier. In smaller α (= $\alpha_{r}\cdot \alpha_{1}$), *C* can be inversely increased by (4). $z_{x}$ extracted from the standard deviation of the received signals can be expressed by (17). By the result of fit errors, the model in (17) well agreed the measured $z_{x}$ with less than 7%. Both the analytical model and the experimental result for $z_{x}$ clearly show that $z_{x}$ varies with respect to *n* and mostly proportional to $\alpha_{r}$. As expected in (13) and (16), the SNRs of *M-H* sequences in $r_{ij}$ and $\left|\Delta r_{ij}\right|$ were determined by *m* and were better than those of *B-H* matrices. However, the conventional *W-H* matrices with *n* = 4 and *n* = 8 as well as *M-H* matrices with n≥9 should lower down $\alpha_{r}$ to satisfy *M*. Their configuration increases $z_{x}/\alpha_{r}$ due to the term of the amplifier noise related to *C* in (17). This also indicates that the SNRs for regular *B-H* matrices are superior than the ones for *W-H* matrices with small $\alpha_{r}$ as reported in [1]. Therefore, we can expect that the inherent regularity of the proposed *M-H* matrices can keep $\alpha_{r}$ high so that the resulting SNR can be improved within the signal capacity *M*.

**Figure 11:**
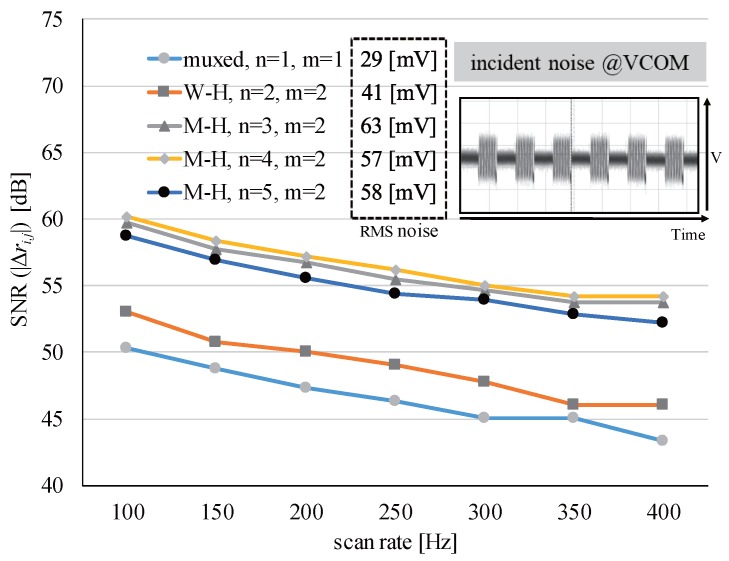
Changes in the SNR according to the scan rate and the interference level.

Figure 11 shows the variations in the SNRs of touch events with respect to the scan rates and their level of interference on the display panel for different *H* matrices. The multiplexed driving of TXs in which only a single TX was active at any time yielded the lowest SNR because it had the shortest sensing time per sensor node. For different time constraints due to the scan rates, small fluctuations were found, but *M-H* matrices with *m* = 2 exhibited almost the same SNRs as expected in (13). They also showed similar interference levels. RMS noise voltages observed in the display common electrode (VCOM) plane depended on *m*. This comes from that the incident driving noise on VCOM is determined by the column sum of the *H* matrix and the parasitic capacitance.

**Figure 12:**
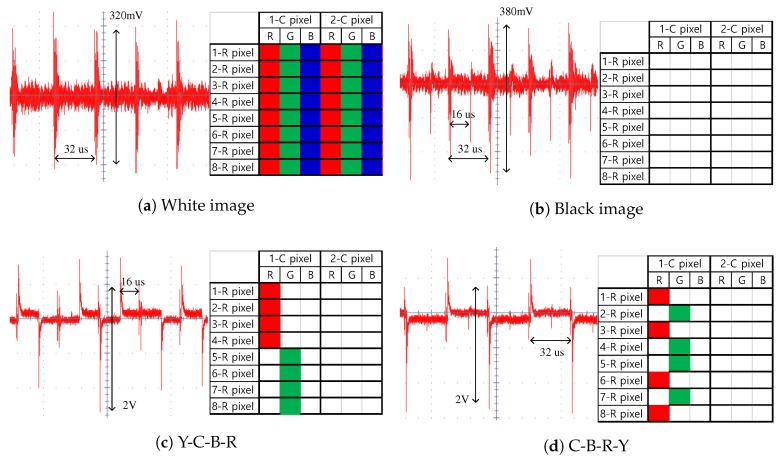
Display noise patterns in eight rows and two adjacent pixel columns induced by four different images; a white blank means “off state”, and all colored squares denote “on state” in RGB sub-pixels.

Figure 12 shows four display noise patterns on capacitive sensors when a two-dot inversion display panel was used [3]. The display driver noise mainly exists in the full-length and the half-length of a horizontal sync (Hsync) period. According to the transfer characteristics shown in Figure 7a, after the de-modulation at the receiver, these noises remained as in-band components.

**Figure 13:**
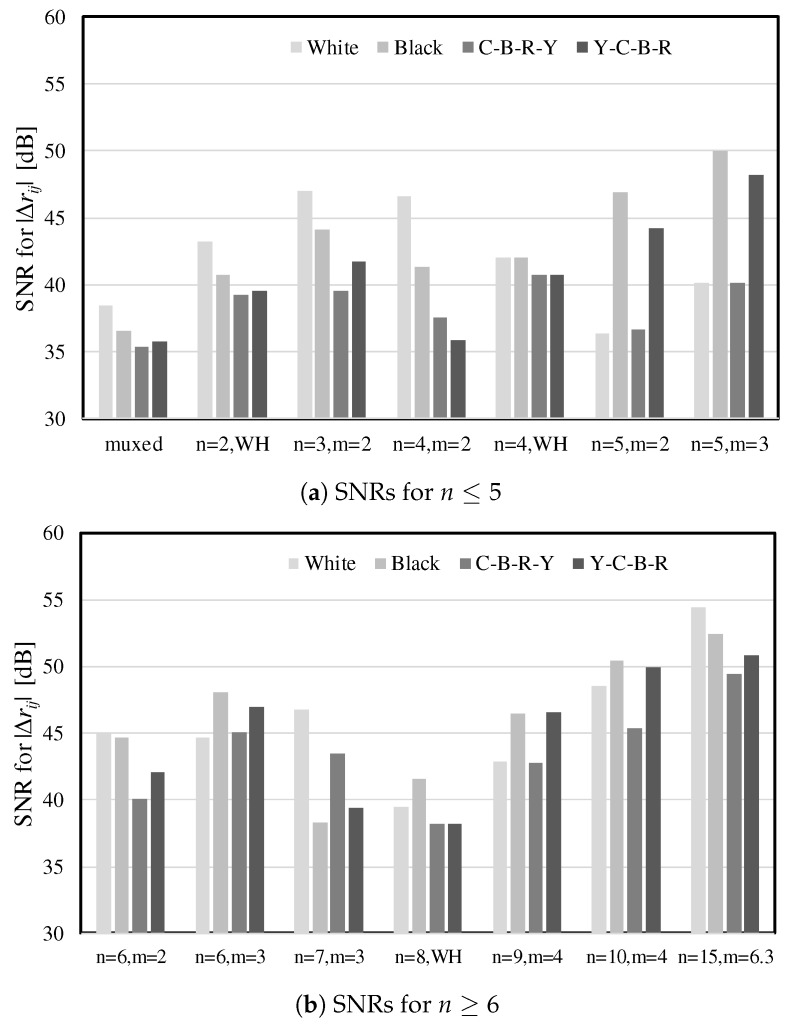
Effects on the SNRs of received signals for the existence of different display noises.

Figure 13 provides the SNR comparison results for the remaining display noise when the worst noise patterns (Y-C-B-R and C-B-R-Y) were displayed below the touch panel. As indicated by (13) and (16), noise immunity for *M-H* matrices are increased by *m* and is more than that of a *B-H* sequence. However, several cases such as *n* = 5 show that less display noise do not always obtain higher SNRs compared to the case of the worst display image. This result was originally caused by the phase difference between $e_{ext}$ and the modulating wave defined in (5), which also makes a difference between the periods of $e_{ext}$ and the integrating samples in the multiple codes. Since the dynamic and accidental noise including display noise is not synchronized to the modulating wave in the sensor, the modulated and accumulated noise values can vary along the code sequence.

To maximize the SNR and the reporting rate under the limited signal capacity *M*, the proposed *M-H* can be a better solution than the existing driving techniques. Table 2 shows another comparative result for SNR and the reporting rate when we try to select the best driving code for entire 15 TX channels of the given touch panel. The SNRs reported in Table 2 can be regarded as those of column-parallel receivers [4,5,9,10,11,27]. Since *n* = 15 is not a multiple of four, in the case of a tuned *H* matrix, an order-16 sequence must be applied for concurrent driving. Under the same maximum column sum *m* = 5, in the *M-H* matrix with *n* = 15, 25% of SNR (≃ 2 dB) can be improved compared to that of the tuned *H* matrix. This result also agreed with our theoretical model in (13) and (16). Apparently, the scan time of the *M-H* matrix which consists of 15 codes in a sequence can be further reduced in the case of a tuned *H* matrix. On the other hand, a fully multiplexed driving method for 15 TX can be represented by the order-15 *M-H* matrix where *a* = *m* and *b* = 0. From (13), *a* should be set to 5 to obtain the same signal gain in the *M-H* matrix. However, if we further limit the maximum TX level as in the *M-H* case, the SNR should be reduced by 6 dB compared to the *M-H* matrix.

## 5. Conclusions

A new driving technique for *M-H* matrices and their effective variants for capacitive touch sensors has been presented. These regular-type matrices produce aggregated signals with the same level at each receiver. They can be configured by changing the column sums, which determine the SNRs of the capacitive signals. The regularity of the capacitive signal reduces the maximum signal capacity of the receiver. This efficiency clearly increases the SNR compared to the conventional *B-H* codes under their limited capacity and the constrained driving strengths. Additionally, the proposed technique enables a higher gain configuration which decreases the amplifier noise. The orders of the matrices can be even or odd, whereas the orders of the conventional *B-H* codes are limited to multiples of four. Therefore, the proposed method supports various numbers of TXs and time constraints so that the SNR can be improved for concurrent signal multiplexing. Resolving the signal integrity issues between the touch and display channels is crucial, and the proposed technique allows for the optimization of SNR and TX levels based on the sensor requirements. Although the *M-H* matrix requires more complex driving circuits than that of the *B-H* codes, they can be implemented by sophisticated and simplified designs if we determine the target number of touch channels and the proper *M-H* sequences.

Other contributions of this work are establishing a system model and defining the SNR for capacitive touch sensors, as the existing communication systems have accomplished a quantitative view. When we specify the budgets of the design parameters, it is possible to estimate SNRs for the *B-H* and *M-H* sequences in a system design stage. The multiplexing technique has been widely used to increase the channel capacity in communication and sensor application systems. Our extensive work focuses on developing a unified system model as well as its implementation techniques for heterogeneous sensors that can be integrated into system-on-chip designs.

## Figures and Tables

**Table 1:** Observed SNRs for $r_{ij}$ and $\left|\Delta r_{ij}\right|$ in *W-H*s [1,10,12,13,14] and proposed *M-H*s where $\alpha V_{TX}≃101$ mV.

Code	SNR for $r_{\mathrm{ij}}$ [dB]	SNR for $\left|\Delta r_{\mathrm{ij}}\right|$ [dB]	Normalized α ($\alpha_{r}$)	Observed $z_{x}$ [mV]	Model for $z_{x}^{2}$	Model Error for $z_{x}$${}^{b}$ [%]
n=1, Fully-muxed	54.5	40.3	1.0	0.191	A+B+C	2.3
n=2, *W-H*	57.2	43.0	1.0	0.199	2A+B+C	2.1
n=3, m=2	60.9	47.7	1.0	0.210	3A+B+C	4.0
n=4, m=2	59.6	47.2	1.0	0.213	4A+B+C	2.1
n=4, *W-H*	58.7	44.7	0.5	0.116	A+0.25B+4C	0.2
n=5, m=3	62.7	49.2	1.0	0.222	5A+B+C	3.1
n=6, m=2	58.8	45.9	1.0	0.220	6A+B+C	0.6
n=6, m=3	60.7	49.2	1.0	0.236	6A+B+C	6.5
n=8, *W-H*	56.7	42.4	0.25	0.122	0.5A+0.0625B+16C	0.7
n=9, m=4 ${}^{a}$	63.8	51.7	0.5	0.133	2.25A+0.25B+4C	1.6
n=10, m=4 ${}^{a}$	63.7	50.9	0.5	0.137	2.5A+0.25B+4C	2.6
n=15, m=6.3 ${}^{a}$	67.8	54.0	0.5	0.139	3.75A+0.25B+4C	5.0
n=15, m=6.6 ${}^{a}$	69.3	54.8	0.5	0.141	3.75A+0.25B+4C	3.5

${}^{a}$ *M-H* matrix expanded using the Kronecker product; ${}^{b}$ Errors were extracted by applying *A* = 0.05 mV${}^{2}$, *B* = 0.18 mV${}^{2}$, *C* = 0.03 mV${}^{2}$.

**Table 2:** Comparative results for a full scan of 15 TX channels.

Code	Max. Column Sum (*m*)	SNR($r_{\mathrm{ij}}$) [dB]	SNR($\left|\Delta r_{\mathrm{ij}}\right|$) [dB]	Scan Rate ^b^ [Hz]	Max. TX [V]
n=15, *M-H* ^a^	5	65.9	52.1	600	4.3
n=15, *M-H* ^a^	4	63.9	50.2	600	4.0
n=16, tuned *H*	5	64.0	50.1	563	3.4
n=1, fully muxed	1	54.5	40.3	600	3.4
n=1, fully muxed	2	60.3	46.1	600	4.3

^a^ constructed by $H_{5}⊗H_{3}$; ^b^ scan rates for single receiver.
